# A respiratory compensating system: design and performance evaluation

**DOI:** 10.1120/jacmp.v15i3.4710

**Published:** 2014-05-08

**Authors:** Ho‐Chiao Chuang, Ding‐Yang Huang, Der‐Chi Tien, Ren‐Hong Wu, Chung‐Hsien Hsu

**Affiliations:** ^1^ Department of Mechanical Engineering National Taipei University of Technology Taipei Taiwan; ^2^ Department of Radiation Therapy and Oncology Shin Kong Wu Ho‐Su Memorial Hospital Taipei Taiwan

**Keywords:** real‐time tracking, compensated respiratory motion, GAFCHROMIC film

## Abstract

This study proposes a respiratory compensating system which is mounted on the top of the treatment couch for reverse motion, opposite from the direction of the targets (diaphragm and hemostatic clip), in order to offset organ displacement generated by respiratory motion. Traditionally, in the treatment of cancer patients, doctors must increase the field size for radiation therapy of tumors because organs move with respiratory motion, which causes radiation‐induced inflammation on the normal tissues (organ at risk (OAR)) while killing cancer cells, and thereby reducing the patient's quality of life. This study uses a strain gauge as a respiratory signal capture device to obtain abdomen respiratory signals, a proposed respiratory simulation system (RSS) and respiratory compensating system to experiment how to offset the organ displacement caused by respiratory movement and compensation effect. This study verifies the effect of the respiratory compensating system in offsetting the target displacement using two methods. The first method uses linac (medical linear accelerator) to irradiate a 300 cGy dose on the EBT film (GAFCHROMIC EBT film). The second method uses a strain gauge to capture the patients' respiratory signals, while using fluoroscopy to observe *in vivo* targets, such as a diaphragm, to enable the respiratory compensating system to offset the displacements of targets in superior‐inferior (SI) direction. Testing results show that the RSS position error is approximately 0.45 ~ 1.42 mm, while the respiratory compensating system position error is approximately 0.48 ~ 1.42 mm. From the EBT film profiles based on different input to the RSS, the results suggest that when the input respiratory signals of RSS are sine wave signals, the average dose (%) in the target area is improved by 1.4% ~ 24.4%, and improved in the 95% isodose area by 15.3% ~ 76.9% after compensation. If the respiratory signals input into the RSS respiratory signals are actual human respiratory signals, the average dose (%) in the target area is improved by 31.8% ~ 67.7%, and improved in the 95% isodose area by 15.3% ~ 86.4% (the above rates of improvements will increase with increasing respiratory motion displacement) after compensation. The experimental results from the second method suggested that about 67.3% ~ 82.5% displacement can be offset. In addition, gamma passing rate after compensation can be improved to 100% only when the displacement of the respiratory motion is within 10 ~ 30 mm. This study proves that the proposed system can contribute to the compensation of organ displacement caused by respiratory motion, enabling physicians to use lower doses and smaller field sizes in the treatment of tumors of cancer patients.

PACS number: 87.19. Wx; 87.55. Km

## I. INTRODUCTION

In cancer treatment, *in vivo* organ movements caused by respiratory motion would result in a considerable impact for physicians in clinical diagnosis and radiation therapy. Many studies have shown that motion artifacts will result from organ displacements caused by respiratory motion in patients' computed tomography (CT). As a result, the physician must rely on other technologies, including four‐dimensional computed tomography (4D CT), to determine the location of the tumor, as well as the field and size for radiation therapy.^1^, ^2^, ^3^, ^4^, ^5^, ^6^, ^7^ A number of research teams have attempted to use fluoroscopy, magnetic resonance imaging (MRI), intensity‐modulated radiation therapy (IMRT), and 4D CT approaches to analyze the displacements of different tumors, organs, and areas (liver, lung, diaphragm) caused by respiratory motion.(8‐17) These studies found that, the superior‐inferior (SI) organ displacement is greater than the anteriorposterior (AP) and medial‐lateral (ML) displacement, and the displacement is greater when closer to the diaphragm.^(18)^

Many research teams have attempted to solve the organ displacement problem by various approaches, including gating^19^, ^20^, ^21^ and breath‐holds technology.^22^, ^23^, ^24^, ^25^ However, the above two methods will cause the lengthening of treatment time and patient discomfort. The real‐time tracking^19^, ^20^, ^26^, ^27^, ^28^ approach requires more expensive equipment; therefore, these approaches are yet to be improved.

In 2012, Buzurovic et al.^(29)^ presented a real‐time couch tracking control technique for 4D tumor tracking by using a commercial treatment couch, and the tumor tracking could be in a single direction or in all three directions. In 2006, D'Souza and McAvoy^(30)^ used an existing treatment couch (Hexapod) to study the couch dynamics, and created an internal model controller to simulate feedback control of respiration‐induced motion. Their motion data were obtained by using a skin marker placed on the abdomen of the patient to track the tumor motion. In 2011, Buzurovic et al.^(31)^ proposed a novel approach to the 4D active tracking and dynamic delivery, incorporating the tumor motion prediction technique. An algorithm was invented to predict the tumor position, and the robotic systems are able to continuously track the tumor during radiation therapy. In 2005, D'Souza et al.^(32)^ constructed a miniature adaptive couch model consisting of two movable platforms that can simulate tumor motion and couch motion. After the initial trial, they proposed a real‐time couch compensation system by using a stereoscopic infrared camera system interfaced to a commercial robotic couch (Hexapod) to address the tumor motion problem. In 2012, Haas et al.^(27)^ presented a couch‐based active motion compensation system. It employed a control strategy that combines a Kalman filter to predict the surrogate motion and, with the control action calculation, the couch position can be estimated.

This study attempts to use a strain gauge to capture the patient's respiratory signals, and a proposed respiratory compensating system for target area displacement compensation. The strain gauge voltage change is the source of respiratory signals to drive the respiratory compensating system in reverse motion to offset organ displacement caused by breathing phenomenon. Coupled with fluoroscopy, this study also analyzes and discusses the compensation effect.

In this study, the proposed respiratory compensating system is mounted on the top of an existing treatment couch. With advantages including simple structure, easy assembly, low cost, easy mobility, and no alteration of the existing treatment room, it can be applicable to radiation therapy machines or CT machines of different systems to reduce the impact caused by organ motion in treatment plans and during treatment to alleviate radiation damage to tumor adjacent tissues.

## II. MATERIALS AND METHODS

### A. System Design

#### A.1 Respiratory signal capture device — strain gauge

**Figure 1:**
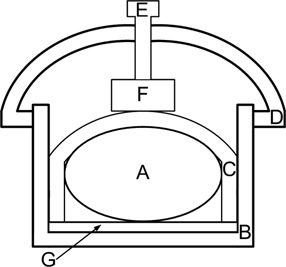
Strain gauge setup diagram. A is a human body (profile), B is the body fix, C is the cast, D is the arch‐type fixture, E is the compression rod, F is the strain gauge, and G is the hip fix.

This study uses a strain gauge (VISHAY SR‐4 precision sensors; Vishay Precision Group, Wendell, NC) device installed inside an arch‐type fixture, which is fixed on the human abdomen. By using the RSS or compression rod to press the strain gauge, as shown in Fig. 1, the strain gauge will deform to change the strain gauge electrical resistance and lead to an imbalance of the internal Wheatstone bridge inside the connected strain amplifier (KYOWA WGA‐670B; Kyowa Electronic Instruments Co. Ltd., Tokyo, Japan); thus, outputting the changing voltage signals is caused by respiratory motion.

#### A.2 Target simulator

**Figure 2:**
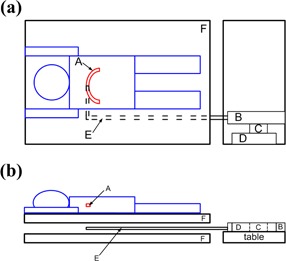
Top view (a) and side view (b) of the target simulator. A is the diaphragm, B is the linear actuator, C is the linear guideway, D is the optical scale, E is the glass fiber tube and L‐type metal rod, and F is the respiratory compensating system.

The target simulator is composed of a linear actuator, linear guideway, optical scale (Linear Encoder), glass fiber tube, and L‐type metal rod, as shown in Fig. 2. The voltage signals are transmitted from the strain amplifier by software (VisSim; Visual Solutions Inc., Westford, MA) to control the displacement of the target simulator, and the mobile fluoroscopy system (Philips C‐arm BV Endura; Philips Healthcare, Andover, MA) is used to observe the displacements of tracking targets (tumor, diaphragm, or other organ) in order to adjust the gain and shift of voltage signals of the strain gauge to further make the displacements of target simulator and the target identical. The shift of voltage signal in this study is only used for the alignment of the diaphragm. Then, the adjusted voltage signals are sent to the respiratory compensating system for reverse motion in the opposite direction of the couch and target displacement to achieve mutually offsetting effects. The respiratory compensating system is only capable of performing compensation in SI direction.

#### A.3 Respiratory compensating system

**Figure 3:**
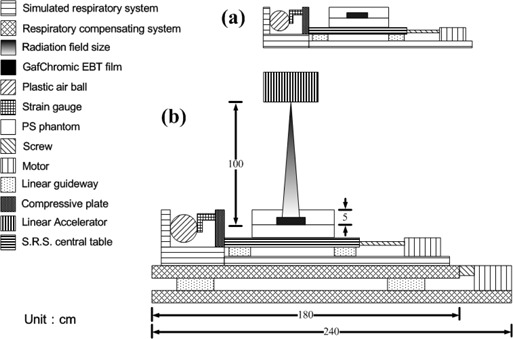
Schematic diagram (a) of the respiratory simulation system (RSS) and (b) of the simulated respiratory and compensating system.

This study employs dose verification and human body experiments for the verification of a respiratory compensating system. In the dose verification, as shown in Fig. 3(a), the polystyrene (PS) phantom is placed on the center table of an RSS system. The dimensions of the PS phantom is 250(L)×250(W)×49.8(T)mm; therefore, the GAFCHROMIC EBT film (International Specialty Products, Wayne, NJ) could be placed at a fixed distance from the linac radiation source. Dose verification measurements are divided into two parts. In the first part, only the RSS system is started to input sine wave or human respiratory signals to generate the simulated organ or tumor displacement, and a medical linac is used to irradiate the EBT film in the PS phantom to obtain the radiation dose distribution on the film in case of respiratory motion. The second part is to fix the strain gauge at the compressive plate at the front end of the RSS system, as shown in Fig. 3(b). This allows the RSS system to push the compressive plate forward and press the strain gauge between the plastic air ball and compressive plate. As a result, slight voltage changes occur in the Wheatstone bridge of the strain gauge. Voltage signals are then transmitted to the strain amplifier, where the amplified voltage changes are sent into a computer to initiate the respiratory compensating system. Then, the linac is used to determine the radiation dose distribution on the EBT film in case of respiratory compensation.

Our proposed respiratory compensating system is only capable of performing compensation in SI direction. However, if the tumor motions in AP and LR directions are compensated in our respiratory compensating system, the total height of the entire equipment is too large to enter the gantry. In addition, the displacement of tumor due to respiratory motion in AP and LR directions is only around 20% of the SI direction. Thus, it will not show a significant improvement in terms of the overall compensating rate if the respiratory motions in AP and LR directions are compensated. Moreover, our proposed respiratory compensating system has a position error of around ±2 mm itself, which is similar to the amount of the displacement of tumor in AP and LR directions. Even if the respiratory motion in AP and LR directions are compensated, the system still causes some position errors.

### B. System testing and calibration

#### B.1 Mobile fluoroscopy system calibration

At present, the fluoroscopy system uses image‐intensifier‐based digital fluoroscopy to convert an invisible X‐ray image into visible image. This process is subject to the impact of terrestrial magnetism and may result in image distortion. Hence, many calibration approaches are required to calibrate the image to conform to the actual situation.^(33)^

**Figure 4:**
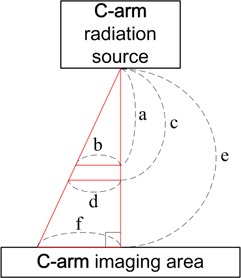
C‐arm calibration by principle of similar triangles.

Figure 4 illustrates target simulator displacement calibration using the mobile fluoroscopy system (C‐arm), following the principle of similar triangles. As shown in the figure, *a* is the distance between C‐arm radiation source and the target (crosswire, diaphragm, hemostatic clip or tumor), *b* is the actual distance of the target movement, *c* is the vertical distance from C‐arm radiation source to L‐type metal rod of the target simulator, *d* is the actual displacement of the L‐type metal rod of the target simulator, *e* is the vertical distance from C‐arm radiation source to C‐arm imaging area, and *f* is the projected movement distance of *b* and *d* after C‐arm radiation source irradiation (the displacements of *b* and *d* in the image after projection amplification are identical). When a, c, e, and f are known, b and d displacements can be calculated by a:b=e:f and c:d=e:f and d/b values can be calculated by b and d displacements. Next, the displacements of the target simulator can be corrected by dividing the value (d/b), and the corrected displacement can be sent to the respiratory compensating system for respiratory displacement compensation.

Since the target simulator L‐type metal rod is in a fixed location of the respiratory compensating system (namely, “c” in Fig. 4 is fixed), in the following human body experiments, *in vivo* targets' displacements (Fig. 4 “b”) at different vertical distances (Fig. 4 “a”) from the radiation source can be inferred by the displacement of the L‐type metal rod (Fig. 4 “d”). Therefore, the compensation of the respiratory compensating system can be more accurate and the phenomenon of overcompensation can be prevented.

### C. Experiment design

#### C.1 The dose distribution experimental method for the respiratory compensation system

This study uses strain gauge as the device to capture the respiratory signals. To verify the effect of the strain gauge in respiratory compensation, the dose verification method is used to quantify the data of the improvement. According to literature,^9^, ^34^ when a tumor is affected by respiratory motion, it may result in tumor displacement in the range of 3.9∼39 mm due to location differences, and the cycle for one breath is between 2.9∼5.6 sec.^(9)^ Therefore, this study selected a cycle of 4 sec as the breathing cycle, and used the sine wave in amplitudes of 5 mm, 10 mm, and 15 mm (in cases of couch displacements of 10 mm, 20 mm, 30 mm) as the RSS input signals. This study also analyzes EBT irradiation dose distribution in the case of using actual human respiratory signals as the RSS signals. Hence, the strain gauge is used to capture human respiratory signals, and the range of moderate respiratory amplitude changes and displacement are selected as the source of respiratory signals. The total time of the obtained human respiratory signals is 60 sec. After the respiratory amplitude adjustment, respiratory signals of amplitudes of about 5 mm, 10 mm, and 15 mm (in the case of couch displacements at 10 mm, 20 mm, 30 mm, respectively), are produced. After obtaining the respiratory signals, this study conducts seven groups of experiments: three uncompensated groups, three compensated groups, and one standstill group. The sine waves at amplitudes of 5 mm, 10 mm, and 15 mm (in the case of couch displacements at 10 mm, 20 mm, 30 mm, respectively) and respiratory signals are inputted into the RSS system for the respiratory simulation and respiratory compensation dose verification experiments.

The original EBT film size is 20.32×25.4 cm, and is cut into six equal parts for discussion of dose distribution under the same field size (10.16×8.382 cm). The linac field size is set as 3×3 cm, and the EBT film is placed in the field size center. The linac energy is set at 10 MV and dose rate was set as 515 MU/min. The EBT film is placed in the PS phantom in the dimensions of 25 cm in length and width, 49.8 cm in thickness, and at a distance of 95 cm from the linac radiation source to surface distance (SSD). It takes 44.4 sec of 11.1 respiration cycles to deliver a dosage 300 cGy.

The PS phantom is placed on the RSS acyclic central table, as shown in Fig. 3(b), and a 5 cm thick PS phantom is placed on the EBT film to measure the compensation effect of the respiratory compensating system. The sine waves of the 4 sec cycle (amplitudes at 5 mm, 10 mm, and 15 mm, respectively, for table displacements of 10 mm, 20 mm, and 30 mm) and human body respiratory signals (for couch displacements of 10 mm, 20 mm, and 30 mm) are used as the RSS input signals. The human body respiratory signals are first recorded by the strain gauge, and then the voltage signals generated from the strain gauge are sent into the target simulator in order to reproduce the human body respiratory signals. Linac is used to irradiate EBT film in the compensated, uncompensated, and standstill states. The dose analysis software (DoseLab; Mobius Medical Systems, Houston, TX) is used to analyze the dose distribution status.

#### C.2 Experimental methods of human body experiments

After the dose verification of the compensation effect on the respiratory compensating system, Shin Kong Wu Ho‐Su Memorial Hospital applied to the Institutional Review Board (IRB) for human body experiments (Application No.: 20120201R), with the consent of the patients and their families. This study uses a mobile fluoroscopy system (C‐arm) to conduct fluoroscopy of the patients' chests in an attempt to use the respiratory compensating system to compensate human organ displacements caused by respiratory motions.

**Figure 5:**
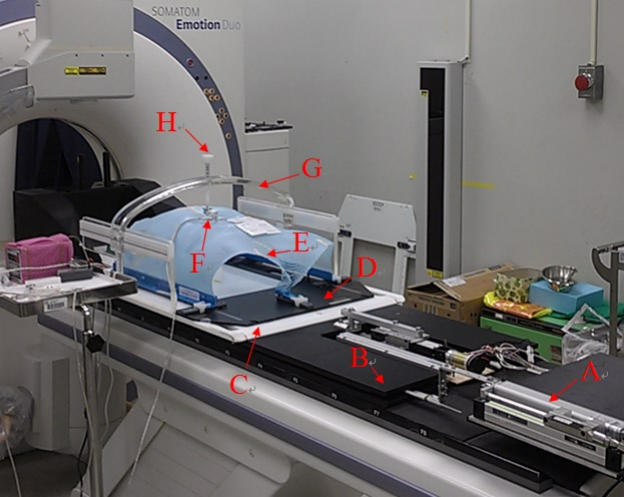
Human body experiment architecture. A is the target simulator, B is the respiratory compensating system, C is body fix, D is the hip fix, E is the thermoplastic cast, F is the strain gauge, G is the arch‐type fixture, and H is the compression rod.

First, the respiratory compensating system is mounted on an existing CT couch of the hospital and uses a target simulator as the aligner of the targets, such as the diaphragm and hemostatic clip. After installing the hip fix in the respiratory compensating system, the patient is asked to lie in the hip fix, and the central position of the body fix is corrected by laser alignment; then the customized thermoplastic cast is used to fix the patient in the body fix, as shown in Fig. 5. After fixing the patient, a strain gauge is placed on the patient's abdomen, and the arch‐type fixture of the body fix is used to connect the compression rod to press the strain gauge. When a patient is breathing, the abdomen goes up and down with the respiratory motion, pressing or relaxing pressure of the compression rod on the strain gauge. The voltage signals can represent human respiratory signals. Then, the voltage signals are input into the target simulator.

This study thus uses the mobile fluoroscopy system (C‐arm) to observe the displacement error relationship between the L‐type metal rod and diaphragm displacement. After recording of the fluoroscopy images, image analysis software (CMA Coach 6; CMA, Amsterdam, The Netherlands) is employed for analysis.

The voltage signal gain and the offset are adjusted during the alignment of the target simulator through the C‐arm. First, the image of C‐arm is observed and the reference position of the target simulator is adjusted to be consistent with the real basis of the diaphragm. Then the signal of the reverse motion in respiratory compensating system can be amplified to drive the motor. After adjusting the voltage signal gain and offset according to this displacement error, the displacements of the L‐type metal rod and target are identical. The adjusted respiratory signals are inputted into the respiratory compensating system to cause the reverse motion in the respiratory compensating system to offset the displacement of the diaphragm caused by respiratory motion.

### D. Data analysis

#### D.1 Displacement error analysis

This study uses the sine wave and actual human respiratory signals as the RSS input signals for the location and compensating error verification of the RSS and respiratory compensating system. Tables 1 and 2 show that, when using the strain gauge as the device to capture the respiratory signals, if the input signals are sine wave, the RSS position error is approximately 0.58∼1.42 mm (with amplitudes of 5, 10, and 15 mm), the respiratory compensating system position error is 0.62∼1.42 mm (with amplitudes of 5, 10, and 15 mm), and the compensating error of the two systems is 0.92∼2.75 mm. If the input signals are human respiratory signals,

**Table 1:** Sine wave movement compensating error

	*Input Signal*		
*Experimental Error (mm)*	*Sine wave amplitude 5 mm*	*Sine wave amplitude 10 mm*	*Sine wave amplitude 15 mm*
RSS position error (mm)	0.58	0.98	1.42
Respiratory compensating system position error (mm)	0.62	1.04	1.42
Compensating error (mm)	0.92	1.65	2.75

**Table 2:** Respiratory motion compensating error

	*Input Signal*		
*Experimental Error (mm)*	*Respiratory displacement 10 mm*	*Respiratory displacement 20 mm*	*Respiratory displacement 30 mm*
RSS position error (mm)	0.45	0.85	1.28
Respiratory compensating system position error (mm)	0.48	0.81	0.87
Compensating error (mm)	1.14	1.70	2.57

RSS position error is 0.45∼1.28 mm (with different displacement of 10, 20, and 30 mm), respiratory compensating system position error is 0.48∼0.87 mm (with different displacement of 10, 20, and 30 mm), and the compensation error of the two systems is 1.14∼2.57 mm.

#### D.2 Profile analysis of dose distribution

**Figure 6:**
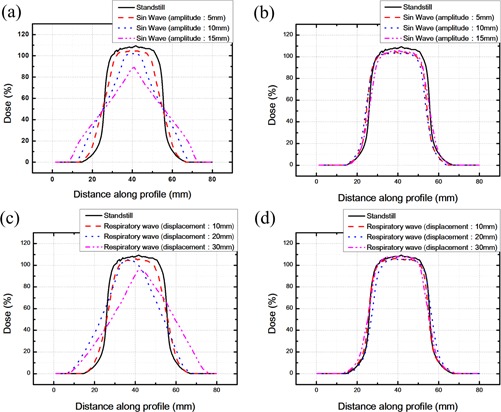
In case of RSS system sine wave or human respiratory signal motions, EBT film profiles based on different inputs of the RSS. Figures (a) and (b) are the EBT film profiles, as based on the sine wave input to the RSS without and with compensation, respectively. Figures (c) and (d) are the EBT film profiles based on the respiratory wave input to the RSS without and with compensation, respectively.

The GAFCHROMIC EBT film is cut into smaller parts, with sizes of 2.54×2.54 cm and was calibrated for analysis of the dose distribution on the EBT film. The dose profile distribution analysis of the GAFCHROMIC EBT film in the horizontal direction along the central line is used to obtain the RSS system motion dose profile diagrams according to sine wave or human respiratory signals. As shown in Fig. 6, the original point of the central horizontal axis refers to the couch side. In the direction of 80 mm is the gantry side. The location at 40 mm is the central point of the film, and 25∼55 mm is the irradiation field size in the standstill state.

#### D.3 Isodose area analysis

**Figure 7:**
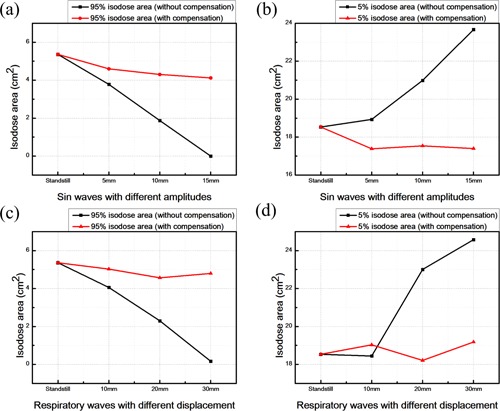
Changes in 95% and 5% isodose areas after the RSS sine wave (a) and (b) or human respiratory signals (c) and (d) movements.

When the couch is at the standstill state, the 95% isodose area is $5.36\;{\text{cm}}^{2}$, 5% isodose area is $18.53\;{\text{cm}}^{2}$. Figures 7(a) and 7(b) illustrate the changes in 95% and 5% isodose areas within the field size region after the sine wave motion of RSS. When the respiratory compensating system is not actuated, the 95% isodose area is reduced by $3.78\;{\text{cm}}^{2}$, $1.87\;{\text{cm}}^{2}$, and $0\;{\text{cm}}^{2}$, while the 5% isodose area is increased to $18.93\;{\text{cm}}^{2}$, $20.99\;{\text{cm}}^{2}$, and $23.67\;{\text{cm}}^{2}$, based on the RSS sine wave movement amplitudes of 5 mm, 10 mm, and 15 mm, respectively. After starting the respiratory compensating system, the 95% isodose area is increased to $4.6\;{\text{cm}}^{2}$, $4.303\;{\text{cm}}^{2}$, and $4.12\;{\text{cm}}^{2}$, while the 5% isodose area is reduced to $17.39\;{\text{cm}}^{2}$, $17.54\;{\text{cm}}^{2}$, and $17.4\;{\text{cm}}^{2}$. When the respiratory compensating system is not started, the 95% isodose area is reduced to $4.06\;{\text{cm}}^{2}$, $2.3\;{\text{cm}}^{2}$, and $0.17\;{\text{cm}}^{2}$, respectively, while the 5% isodose area is increased to $18.44\;{\text{cm}}^{2}$, $23\;{\text{cm}}^{2}$, and $24.58\;{\text{cm}}^{2}$, based on the RSS respiratory wave movement displacement being 10 mm, 20 mm, and 30 mm, respectively. When the respiratory compensating system is started, the 95% isodose area is increased to $5.03\;{\text{cm}}^{2}$, $4.57\;{\text{cm}}^{2}$, and $4.8\;{\text{cm}}^{2}$, while the 5% isodose area is reduced to $19.03\;{\text{cm}}^{2}$, $18.21\;{\text{cm}}^{2}$, and $19.18\;{\text{cm}}^{2}$, as shown in Figs. 7(c) and 7(d).

#### D.4 Gamma analysis within the field size

**Figure 8:**
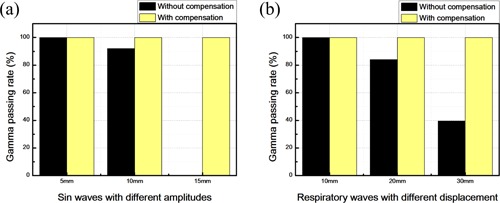
Gamma passing rate analysis with and without compensation: (a) sine waves with different amplitudes input to the RSS; (b) respiratory waves with different amplitudes input to the RSS.

The gamma passing rate is an analysis method used to evaluate the relationship between displacement and dose. The general parameter is 3%/3 mm.^(35)^ If the point passes the dose test, the record is 0, otherwise, it is 1. As shown in Fig. 8(a), when the RSS sine wave movement amplitudes are 5 mm, 10 mm, and 15 mm, respectively, and when the respiratory compensating system is not operated, the gamma passing rates are 100%, 92%, and 0%, respectively. When the respiratory compensating system is started, the gamma passing rate is 100% in all cases, as shown in Fig. 8(b).

#### D.5 Human body verification experiments of the respiratory compensating system

This study aims to verify the impact of a respiratory compensating system on the human body. With the consent of the patients and their families, this study conducts human body verification experiments of the respiratory compensating system. The average age of the patients in this study is 64 years old, the average height is 167 cm, and the average weight is 65 kg. Seven males and three female patients participate in the human body verification assessment of the respiratory compensating system. Regarding the tracking targets, this study tracks the diaphragms of four patients and the *in vivo* hemostatic clip of one patient.

**Figure 9:**
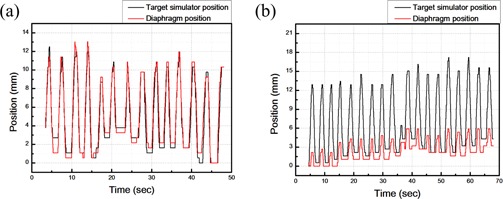
A comparison (a) of the locations of target (diaphragm) and target simulator before the respiratory compensating system is started; (b) the comparison of the locations of target (diaphragm) and target simulator after the respiratory compensating system is started.

An image analysis software, CMA Coach 6, is used to read out the shooting film from the C‐arm to enable the user to click on the desired point on the image (the apex of the diaphragm is usually selected as the target). Then the movement of the diaphragm can be depicted according to the recorded location of clicks. In addition, a ruler is placed in the C‐arm to determine the real scale of the movement of the diaphragm. According to the results of the target location, as analyzed by the CMA Coach 6, before starting the respiratory compensating system, this study obtains the location data of the target simulator and targets (diaphragm and hemostatic clip), which are depicted in the location comparison diagram, as shown in Figure 9(a). The Pearson product‐moment correlation coefficient is used to calculate the correlation between the target simulator and target actual location. The results confirm that the target simulator and *in vivo* organ displacement are correlated. Then, this study initiates the respiratory compensating system. Target simulator displacement is regarded as the displacement of the target (diaphragm, hemostatic clip). This study also conducts a comparative analysis of the compensated target (diaphragm, hemostatic clip) displacement and target simulator displacement, as shown in Fig. 9 and Table 3.

**Table 3:** Human body verification results

*Patient Code*	*Target*	*Correlation of target and target simulator (%)*	*With compensation, target simulator maximum displacement (mm)*	*With compensation, target simulator minimum displacement (mm)*	*With compensation, target simulator average displacement (mm)*	*With compensation, target maximum displacement (mm)*	*With compensation, target minimum displacement (mm)*	*With compensation, target average displacement (mm)*	*Compensation rate (%)*
1	Diaphragm	90.4	8.9	6.4	7.67±0.73	4.3	0.4	1.92±0.92	75.0±12.0
2	Diaphragm	95.7	8.3	2.6	6.05±1.32	3.5	0.4	1.60±0.8	73.6±13.2
3	Diaphragm	98.5	36.1	15.7	21.39±5.7	9.6	2.2	7.00±1.99	67.3±9.3
4	Diaphragm	98.5	12.2	1.8	9.50±1.88	3.5	0.9	2.44±0.62	74.3±6.5
5	Hemostatic Clip	96.5	12.4	4.6	9.23±2.19	4.1	0.8	2.25±0.81	75.6±8.8
6	Diaphragm	98.7	33.5	12.4	23.51±6.8	13.9	0.5	4.10±2.82	82.5±12.0
7	Diaphragm	99.6	27.2	13.4	19.9±3.9	8.7	0.5	3.98±1.93	80.0±9.7
8	Diaphragm	96.0	27.4	11.3	21.81±5.25	13.3	1.1	6.05±2.35	72.27±10.78
9	Diaphragm	95.8	28.3	15.2	22.99±3.49	9.8	1.1	4.88±2.42	78.79±10.5
10	Diaphragm	96.4	18.1	10.6	13.45±1.69	5.3	0.4	3.01±1.23	77.62±11.7

## III. RESULTS

Our displacement error analysis indicated that the cause of the compensating error being greater than the position error is that the respiratory compensating system must press the strain gauge according to the displacement of RSS in order to generate corresponding voltage signals as the input signals of the respiratory compensating system to drive the respiratory compensating system. The process requires 47 ms, and results in the movement of the crosswire on RSS before its compensation; hence, compensating error will be slightly greater than the position errors of the two systems.

The dose distribution results of the RSS and respiratory compensating system are presented in Fig. 6. Figures 6(a) and 6(c) shows that when the respiratory compensating system is not actuated (uncompensated), the slopes of the dose profiles within the field size region are greater than the slopes of dose profiles after sine wave or respiratory wave movements (as shown by the solid lines in Fig. 6), which indicate that the film will receive more unnecessary irradiation dose outside the region of field size after the movement of the RSS couch. If the movement distance is greater, the dose received in the neighboring regions is also greater, which increases damage to the organs surrounding the tumor of the patient under radiation therapy. However, after the respiratory compensating system is actuated (compensated), it can be found that, regardless of sine wave or human respiratory signal movements of the RSS couch, the respiratory compensating system can effectively offset film displacement, rendering the dose received in the field size on the film after the motion is close to the dose received in the standstill state, as shown in Figs. 6(b) and (d).

Based on the results of isodose area analysis, when the RSS system displacement is greater, the isodose area of lower dose (5%) is increased, while the isodose area of higher dose (95%) is declined. After the respiratory compensating system is started, it leads to opposite results. In addition, the gamma analysis results show that, when the RSS respiratory wave displacements are 10 mm, 20 mm, and 30 mm, respectively, and when the respiratory compensating system is not started, the gamma passing rates are 100%, 84%, and 39.6%, respectively. If the respiratory compensating system is started, the gamma passing rate is 100% in all cases.

From the results of the human body verification experiments, as shown in Table 3, it is shown that the target simulator and target correlation is 90.4%∼99.6%, and after the respiratory compensating system is started, the compensation rate is 67.3%∼82.5%. Therefore, the human body verification experiments prove that the proposed respiratory compensating system can effectively reduce organ displacement caused by respiration, and can help physicians to reduce field size and treatment dose.

## IV. DISCUSSION & CONCLUSION

In previous reported studies, all the research teams compensate the tumor motion by proposing a new control strategy or algorithm based on a commercial treatment couch^27^, ^29^, ^30^, ^31^, ^32^ and most of the studies can compensate the tumor motion in three directions. Buzurovic et al.^(31)^ proposed a novel approach to the 4D active tracking and their results showed that the maximum tracking error was within 1 mm. Haas et al.^(27)^ presented a couch‐based active motion compensation system and their tracking errors were between 0.5 and 2 mm. D'Souza and McAvoy^(30)^ used an existing treatment couch to study the couch dynamics, and a skin marker was placed on the abdomen of the patient to track the tumor motion. The resulting residual motion of their compensating system was < 3 mm. Buzurovic et al.^(29)^ revealed that real‐time tumor tracking was feasible using the existing robotic couch with some modifications in control systems. Their couch performance tests show that the motion accuracy of 0.1, 0.1, and 0.12 mm in X, Y, and Z directions, respectively.

In this study, all the components, devices, RSS, and respiratory compensating system are designed and fabricated in‐house. A strain gauge is used as the respiratory signal capture device to capture the simulation and human respiratory signals, which results in the RSS sine wave simulated motion's compensating error in the range of 0.92∼2.75 mm. By comparison with the respiratory compensating system using a pressure bag as the respiratory signal capture device,^(36)^ the improvement of the compensating error can be raised up to 58.9%. In the case of human respiratory signal motion, the compensating error is 1.14∼2.57 mm. The error of 2.57 mm is the measured displacement error between the target simulator and the respiratory compensating system. This means there will be a displacement of 2.57 mm that cannot be compensated by the respiratory compensating system. It will cause some areas of the tumor to be unable to receive the radiation dose, which reduces the compensation rate. By comparison with the respiratory compensating system using pressure bag as the respiratory signal capture device,^(36)^ compensating error can be reduced by up to 61.1%.

This study uses the dose verification method, linac, to irradiate GAFCHROMIC EBT film, and found that the 95% isodose area can be improved by 15.3%∼86.4%, while the 5% isodose area can be reduced by 3.2%∼33.8% with a respiratory compensating system. Regarding human body verification, image analysis found that the correlation between the target simulator and the target can be up to 99.6%, and the maximum compensation rate of the respiratory compensating system can be up to 82.5%. The compensation rate is defined as the compensated displacement of the respiratory compensating system divided by the displacement of the diaphragm due to respiratory motion. This proves that respiratory compensating system can be used to offset the organ displacement caused by respiration, enabling physicians to use lower doses and smaller field sizes in the treatment of tumors of cancer patients. Hence, the *in vivo* normal organ and tissues will suffer less irradiation dose, which may improve postoperational quality of life and survival.

## ACKNOWLEDGMENTS

The authors would like to express their appreciation to the Shin Kong Wu Ho‐Su Memorial Hospital, Taipei, Taiwan for providing the financial and facilities support for this study. The ethical approval is approved by the Shin Kong Wu Ho‐Su Memorial Hospital under the reference number: IRB 20120201R.
